# Long-term Visual Outcomes after Release from Protocol in Patients who Participated in the Inhibition of VEGF in Age-related Choroidal Neovascularisation (IVAN) Trial

**DOI:** 10.1016/j.ophtha.2020.03.020

**Published:** 2020-09

**Authors:** Rebecca N. Evans, Barnaby C. Reeves, Dawn Phillips, Katherine Alyson Muldrew, Chris Rogers, Simon P. Harding, Usha Chakravarthy, IVAN Study Group

**Affiliations:** 1 Clinical Trials and Evaluation Unit, Bristol Trials Centre, Bristol Medical School, University of Bristol, Bristol, United Kingdom; 2 Queen’s University of Belfast, Royal Victoria Hospital, Belfast, Ireland; 3 Department of Eye and Vision Science, Institute of Ageing and Chronic Disease, University of Liverpool, Liverpool, United Kingdom

**Keywords:** CI, confidence interval, DVA, distance visual acuity, ETDRS, Early Treatment Diabetic Retinopathy Study, IQR, interquartile range, IVAN, Inhibition of VEGF in Age-related choroidal Neovascularisation, nAMD, neovascular age-related macular degeneration, RCT, randomized controlled trial, SD, standard deviation, VA, visual acuity, VEGF, vascular endothelial growth factor

## Abstract

**Purpose:**

To describe visual outcomes, frequency of treatment and monitoring visits, and anti–vascular endothelial growth factor drugs used in usual care in participants who exited a trial in which treatment for neovascular age-related macular degeneration (nAMD) was initiated with bevacizumab or ranibizumab.

**Design:**

Multicenter cohort study up to 7 years after trial exit.

**Participants:**

Patients enrolled in the Inhibition of VEGF in Age-related choroidal Neovascularisation (IVAN) trial; after excluding participants from 2 sites and who died or withdrew during the trial, 537 were included in this follow-up cohort.

**Methods:**

Data were collected between May 26, 2016, and August 24, 2017. Distance visual acuity (DVA) (letters read) in both eyes and treatments for nAMD administered to either eye at all usual care visits were extracted from medical records of all participants until the point of data collection (duration of study eye monitoring).

**Main Outcome Measures:**

Rate of change of DVA during active surveillance of the study eye (study eye monitoring), estimated using a multivariable linear random effects model. Other outcome measures were visit and treatment frequency and switches in anti–vascular endothelial growth factor (VEGF) drug.

**Results:**

Data were obtained for 99% (532/537) of eligible participants. The median duration of study eye monitoring after IVAN exit was 3.3 years (interquartile range [IQR], 1.3–4.7), and median DVA was 58.0 letters (IQR, 34.0–73.0). Study eye DVA deteriorated by 4.3 (95% confidence interval [CI], 3.7–4.9) letters per year. Injection rate did not influence the rate of change in DVA after adjusting for key covariates. After IVAN exit, 174 participants (32%) received no treatment; 332 of 358 (93%) were treated first with ranibizumab, 78 (23%) of whom switched to aflibercept. The DVA was similar among participants who switched or did not switch at the end of study monitoring.

**Conclusions:**

Approximately 5 years after the IVAN study finished, with unprecedented completeness of follow-up for such a trial, the trajectory of functional decline in the study eye was shown to be greater than that previously reported for incomplete trial cohorts. Anti-VEGF injection rates and treatment switches were not important factors in determining visual acuity outcomes.

The current standard of care for managing neovascular age-related macular degeneration (nAMD) is treatment with biological molecules that bind or suppress anti–vascular endothelial growth factor (VEGF) therapies. Commonly used agents are ranibizumab, bevacizumab, and aflibercept. The benefits of these agents over 2 years of treatment have been thoroughly evaluated in well-designed randomized controlled trials (RCTs).

Outcomes beyond 2 years, on average, at 5 and 7 years after release from protocol,^1^, ^2^, ^3^ have been characterized by follow-on studies of participants previously enrolled in RCTs. However, these reports are subject to bias because of incomplete follow-up, for a variety of reasons: the age of patients with nAMD, which is approximately 80 years, the associated high levels of morbidity and mortality, and patients’ ability to pay.^4^

Data from real world datasets also show that more than two-thirds of the patient population are no longer under review by an ophthalmologist 4 years after starting treatment.^5^, ^6^, ^7^ There is broad agreement that visual acuity (VA) progressively worsens over time. However, because of the selected nature of the patients for whom data are available, the extent of the decline is likely to have been underestimated in reports of outcomes in subgroups of study populations who have continued to attend for treatment.^5^, ^6^, ^7^

Universal access to care in the United Kingdom’s National Health Service provides an opportunity to combine the advantage of characterizing long-term outcomes in a phenotypically well-defined trial cohort^8^ with the advantage of complete follow-up, minimizing bias. In this report, we describe the visual outcomes, monitoring and treatment frequency, cessation of treatment in the study eye, and survival for up to 5 years in participants who were enrolled in the IVAN clinical trial.

## Methods

The IVAN trial findings and detailed protocol have been described.^8^ Briefly, this trial was conducted in 23 sites across the United Kingdom and enrolled 610 participants with a diagnosis of treatment naïve nAMD. A factorial design was used with study eyes randomized to 1 of 2 anti-VEGF drugs ranibizumab or bevacizumab, and monthly versus treatment as needed.^8^ After release from the IVAN protocol (last participant released from protocol in October 2012), all patients were managed within the National Health Service with free access to health care. The IVAN trial,^8^ including this follow-up study, is covered by the original trial registration (ISRCTN92166560) and approved by the office of the research ethics committees Northern Ireland (07/NIR03/37). Participants’ written informed consent at enrollment in the IVAN trial allowed for continued follow-up through collection of clinical data after participants exited the trial but not for any additional research visit.

For the follow-up study,^9^ we invited all surviving participants who had not withdrawn from the trial to confirm their consent for passive collection of data from their medical records. Participants were also invited to attend an extra study visit. Ethics approval was obtained for passive collection of data for participants who had died during the follow-up period (15/NI/0177). We also asked surviving IVAN participants if they wanted to withdraw their original consent, that is, precluding passive data collection. Those who attended the research visit gave written informed consent for the extra visit at the time of attendance.

The research adhered to the principles of the Declaration of Helsinki. An independent study steering committee appointed by the funder had oversight of the scientific integrity of the follow-up study.

### Study Design

The IVAN follow-up study is a multicenter cohort study of all participants who had completed the IVAN clinical trial and who had not withdrawn their consent.

### Study Population

The eligible study population for the IVAN follow-up study comprised 537 of the original 610 participants who completed 24 months of follow-up, had not withdrawn, and had been treated at a center participating in the follow-up study.

### Outcome Measures

The primary outcome was the rate of change in distance visual acuity (DVA) per year during usual care monitoring and treatment of the study eye during follow-up (duration of study eye monitoring), based on all the routinely collected measurements. These measurements were not made to a mandated protocol with refraction. Thus, we distinguish DVA from best-corrected distance visual acuities (BCVA), the latter having been obtained during the IVAN trial or at the research visit for this follow-up study. Other outcome measures were frequencies of visits and injections during study eye monitoring, retinal morphology from the most recent retinal images available (to be reported elsewhere), and length of survival.

### Data Collected

Information extracted from medical records included dates of all usual care visits, DVA at every visit, and anti-VEGF drug used on release from protocol and any subsequent changes in treatment along with the relevant dates. Generic health status using the EuroQol EQ-5D-5L^10^ questionnaire, a secondary outcome in the IVAN trial, was administered at research visits or by post (nonattenders).

### Study Eye Monitoring and Duration of Follow-up

Duration of study eye monitoring after release from protocol was defined as the period of active surveillance of the study eye only up to the time of data collection. Study eye monitoring was considered as having stopped if the patient was described in the medical record as having been discharged from the participating hospital clinic into community care or if treatment was only being administered to the contralateral eye. This latter definition was applied when the date of the most recent injection to the study eye was 1 year or more before the most recent usual care visit and more recent injections were administered only to the fellow eye. The duration of study eye monitoring was not revised if treatment was restarted in the study eye after discharge or after study eye monitoring had been discontinued. All visits and injections were counted during the period of study eye monitoring. If injections were recorded with intervals <25 days, these were assumed to be recording errors and merged with the intervention assigned to later of the 2 dates.

### Statistical Analyses

Demographics were summarized for the entire cohort. Continuous data are summarized by means and standard deviations (SDs) or median and IQR if the distribution is skewed. Categoric data are summarized as numbers and percentages. Time to end of study eye monitoring or censoring (when information was collected for the study) and time to other events were summarized in Kaplan–Meier graphs. The DVAs recorded in logarithm of the minimum angle of resolution or Snellen fraction were mapped to Early Treatment Diabetic Retinopathy Study (ETDRS) letters as shown in Table S1 (available at www.aaojournal.org). The accuracy of DVA recorded during usual care was compared with BCVA when the latter were acquired within 60 days of the date of the DVA measurement.^11^ Annual monitoring visits and injection rates in the study eye were calculated for the duration of study eye monitoring. The number of injections and monitoring visits were summarized for the cohort as a whole and by BCVA category at IVAN exit, for each year after release from protocol. Scatterplots of number of injections and change in DVA for each year after release from protocol were generated. Information describing the type of anti-VEGF drug used at release from protocol, switches in the drug used during study eye monitoring, time to switch, and DVA at switch is summarized. For study eyes that did not switch, we estimated the predicted median DVA at the median time to switch from the mixed effects model described next.

To estimate the average change in DVA, we fitted a multivariable mixed effects regression model using all available DVA measurements between release from protocol and end of study eye monitoring. We did not include the BCVA measurement made at the research visit. The following covariates were fitted: age at IVAN exit, sex, index of multiple deprivation (IMD),^12^ presence of nAMD in fellow eye at IVAN exit, study better than fellow eye at IVAN exit (defined as study eye BCVA ≥5 letters than fellow eye), injection rate in study eye, and proportion change in lesion size between IVAN entry and IVAN exit.

Injection rate in the study eye was treated as a time-dependent variable. For any given year, the rate of injection for the preceding year was included in the model, estimating the effect of injection rate in the preceding year on DVA in the subsequent year. Further details of the statistical methods are given in the Supplementary material (available at www.aaojournal.org).

Three sensitivity analyses were carried out: (1) excluding DVA measurements during the first year of follow-up; (2) including DVA measurements between the end of study eye monitoring and last recorded usual care visit; and (3) excluding study eyes with missing covariates. Justifications for these analyses are described in the Supplementary material (available at www.aaojournal.org).

Methods for the analyses by original trial allocations, that is, ranibizumab versus bevacizumab and monthly versus as needed treatment, are described in the Supplementary material (available at www.aaojournal.org). All analyses were performed using Stata version 15.1 (StataCorp LP, College Station, TX).

## Results

### Description of Cohort

Data collection from usual care medical records and research visits occurred between May 26, 2016, and August 24, 2017. The cohort comprised 537 patients who were alive and who had not withdrawn at IVAN exit at sites that participated in the IVAN follow-up study. The flow of participants during follow up is shown in Figure S1 (available at www.aaojournal.org). Five participants withdrew their consent for passive data collection. We obtained data for all the remaining 532 participants (99%) who were eligible for inclusion; 413 were still alive, and 199 attended a research visit. Demographic characteristics, medical history, and blindness registrations by original trial allocations are shown in Tables S2 to S4 (available at www.aaojournal.org).

### Duration of Study Eye Monitoring

Time to end of study eye monitoring is shown by BCVA category at IVAN exit in Figure S2 (available at www.aaojournal.org). Only eyes in the worst VA category (<37 letters) at IVAN exit were monitored for substantially less time. The status of study eyes at the end of study eye monitoring was: monitoring continuing 41%; monitoring of fellow eye only 7%; discharged 28%; deceased 14%; other (e.g., care transferred to a nonparticipating hospital) 9%. The median duration of study eye monitoring was 3.3 years (interquartile range [IQR], 1.3–4.7 years). The numbers of clinic visits and intravitreal injections administered during study eye monitoring are shown in Table S5 (available at www.aaojournal.org).

### Distance Visual Acuity at End of Study Monitoring

In the entire cohort, the median BCVA at the IVAN exit visit was 72.0 letters (IQR, 56.0–80.0), and median DVA at the end of study eye monitoring was 58.0 letters (IQR, 34.0–73.0), indicating a loss of approximately 14 letters (Table 1). The median change from BCVA at IVAN exit to DVA at end of study eye monitoring was less −10 letters (IQR, −22.0 to −2.0). At the end of study eye monitoring, one third (35%) had a VA better than 68 letters. Approximately one-fifth (20.8%) had a VA of worse than 33 letters (20/200), and half of these (8.8%) had a VA worse than 18 letters, representing severe vision loss. The change in DVA during study eye monitoring is shown by BCVA category at IVAN exit in Figure S3 (available at www.aaojournal.org).

**Table 1:** Visual Acuity in Study Eyes at IVAN Entry and Exit and End of Study Eye Monitoring

Distance Visual Acuity (Letters)∗	Overall (n = 532)	
	N	%
IVAN entry (BCVA)		
Median (IQR)	65.0	(52.0–74.0)
≤17	0/532	0.0%
18–37	51/532	9.6%
38–52	83/532	15.6%
53–67	169/532	31.8%
68–82	202/532	38.0%
≥83	27/532	5.1%
IVAN exit (BCVA)		
Median (IQR)†	72.0	(56.0–80.0)
≤17	5/530	0.9%
18–37	57/530	10.8%
38–52	49/530	9.2%
53–67	95/530	17.9%
68–82	234/530	44.2%
≥83	90/530	17.0%
End of study eye monitoring (DVA)		
Median (IQR)‡	58.0	(34.0–73.0)
≤17	44/499	8.8%
18–37	95/499	19.0%
38–52	71/499	14.2%
53–67	115/499	23.0%
68–82	150/499	30.1%
≥83	24/499	4.8%

BCVA = best-corrected visual acuity; DVA = distance visual acuity; IQR = interquartile range; IVAN = Inhibition of VEGF in Age-related choroidal Neovascularisation.

∗ All measurements at IVAN entry and exit were BCVA. Measurements at end of study eye monitoring were clinic records of distance visual acuity.

† Data missing for 2 patients.

‡ Data missing for 33 patients.

### Validation of DVA versus Best-Corrected Visual Acuity

Of the usual care DVA measurements, 87% were measured on ETDRS charts and recorded as letter scores (66%) or in logarithm of the minimum angle of resolution notation (21%). Snellen fractions constituted 11%, and hand movements, perception of light, and no perception of light constituted 2%. The Bland–Altman plot in Figure S4 (available at www.aaojournal.org) of the mean difference in DVA and BCVA (DVA minus BCVA) among those who attended the research visit within 60 days of their most recent monitoring visit (n = 95/199) was −1.8 letters (95% confidence interval [CI], −4.3 to 0.6), and the average interval between the measurements was 21 days (standard deviation, 23.8).

### Description of Visit and Injection Rates during Study Eye Monitoring

**Figure 1:**
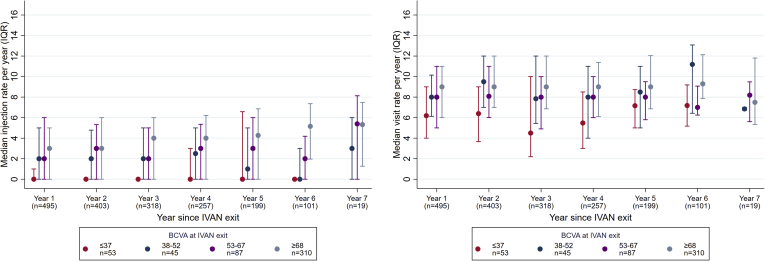
Median injection (left) and visit rate (right) per year since Inhibition of VEGF in Age-related choroidal Neovascularisation (IVAN) exit by best-corrected visual acuity (BCVA) category at IVAN exit. Patients with no appointments since IVAN exit (n = 26) and patients with no visual acuity (VA) since IVAN exit (n = 7) are excluded. Patients monitored for less than 28 days during any given year are excluded from that years’ summary. Pairs of injections that were recorded within 25 days of each other were assumed to be recording errors and merged with the intervention assigned to later of the 2 dates (n = 57 pairs). The best-corrected visual acuity (BCVA) at IVAN exit is missing for n = 2 patients. IQR = interquartile range.

**Figure 2:**
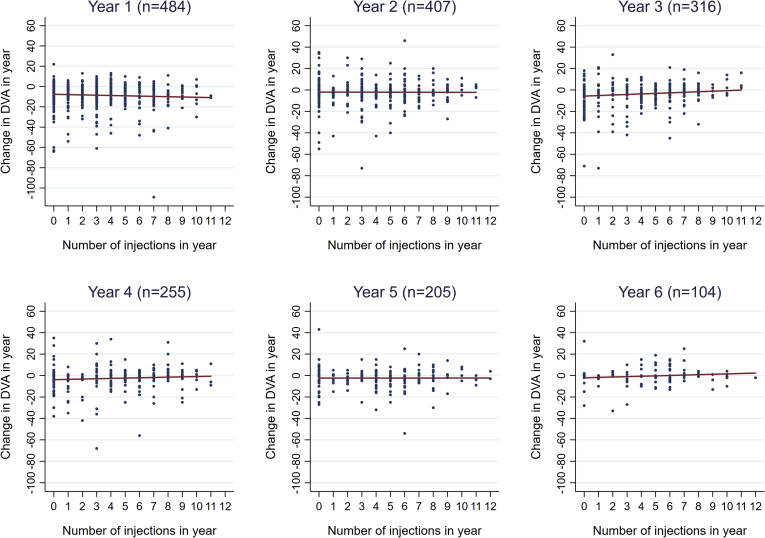
Scatterplots of change in distance visual acuity (DVA) in any given year of follow-up by the number of injections in that year. n = number of patients monitored during each year with at least 1 DVA reading in that year and in the previous year (to calculate change). Pairs of injections that were recorded within 25 days of each other were assumed to be recording errors and merged with the intervention assigned to later of the 2 dates (n = 57 pairs).

A total of 5695 injections were recorded. Rates of monitoring visits and injections are shown for each year of follow-up in Figure 1 and Table S6 (available at www.aaojournal.org). When classified by BCVA category at the IVAN exit visit, the lowest category (≤37 letters) had fewer visits compared with visit rates for the other BCVA groups, which were all similar. Injection rates in the first 4 years of follow-up showed the same pattern, higher for the best 3 categories of BCVA at IVAN exit and lowest in the worst BCVA category. In year 5, the injection rate decreased in the BCVA category of 38 to 53 letters and became similar to that of the worst category. For the entire cohort, the number of injections administered to the study eye was unrelated to the change in DVA in that year (Fig 2).

### Switches in Treatment and Change in Distance Visual Acuity by Switch Status

**Figure 3:**
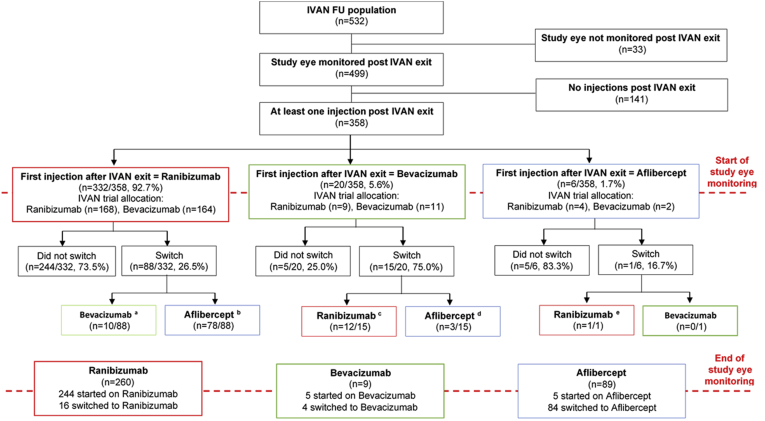
Flowchart of treatment switches after Inhibition of VEGF in Age-related choroidal Neovascularisation (IVAN) exit. ^a^Seven of 10 patients switch again to aflibercept (n = 2); to ranibizumab (n = 2); to ranibizumab then aflibercept (n = 3). ^b^A total of 14 of 78 patients switch again to bevacizumab (n = 1); to ranibizumab (n = 5); to ranibizumab then aflibercept (n = 8). ^c^Five of 12 patients switch again to aflibercept (n = 1); to aflibercept then ranibizumab then aflibercept (n = 1); to bevacizumab then aflibercept (n = 1); to bevacizumab then ranibizumab (n = 1); to bevacizumab then ranibizumab then aflibercept (n = 1). ^d^One of 3 patient switches again to ranibizumab (n = 1). ^e^One of 1 patient switches again: to aflibercept (n = 1). FU = follow-up.

The first anti-VEGF drug administered after release from protocol and subsequent switches in therapy are shown in Figure 3. The majority of study eyes were commenced on ranibizumab at IVAN exit, and few were on bevacizumab or aflibercept. During study eye monitoring, the majority of switches were from ranibizumab to aflibercept (Fig S5, available at www.aaojournal.org). Approximately one-third (174) of study eyes did not receive any further treatments during follow-up, and the median DVA in this group at end of study eye monitoring was 58.0 (IQR, 33.0–73.0) with a median follow-up of 1.0 year (IQR, 0.1–2.2).

Of the 358 participants who continued to receive treatments on release from protocol the majority (93%, n = 332) were commenced on ranibizumab. During follow-up, 78 participants were switched from ranibizumab to aflibercept and 10 were switched to bevacizumab. Demographic characteristics and medical history by study eyes that switched versus those that did not switch are described in Table S7 (available at www.aaojournal.org). The median time to switch was 2.7 years, and the median time from switch to end of follow-up was 2.3 years (Table 2). The median BCVA in the study eye at IVAN exit was similar in switchers at 77 letters (IQR, 70.0–83.0) compared with non-switchers at 73 letters (IQR, 59.5–80.0). At the time of switch, the median DVA in study eyes that switched treatment was 64 letters (IQR, 54.5–73.0). In study eyes that did not switch, the predicted median DVA at the median time to switch (2.7 years) was 61.2 letters. At the end of study eye monitoring, median DVA was 60 letters (IQR, 44.0–69.0) in study eyes that switched and 58 letters (IQR, 33.0–73.0) in study eyes that did not switch.

**Table 2:** Time to Treatment Switch and Distance Visual Acuity According to Whether Treatment Was Switched

Characteristic	Did Not Switch‖ (n = 277)		Switched Treatments‖ (n = 81)		No Treatment Since IVAN Exit (n = 174)		Overall (n = 532)	
	Median	IQR	Median	IQR	Median	IQR	Median	IQR
Time (yrs)								
IVAN exit to switch			2.7	(2.0–3.5)				
Switch to end of SE monitoring			2.3	(1.6–2.8)				
IVAN exit to end of SE monitoring	3.5	(1.7–4.7)	5.0	(4.4–5.7)	1.0	(0.1–2.2)	2.9	(1.1–4.7)
DVA								
IVAN exit∗	73.0	(59.5–80.0)	77.0	(70.0–83.0)	67.0	(39.0–77.0)	72.0	(56.0–80.0)
Date of switch†¶			64.0	(54.5–73.0)				
End of SE monitoring‡	58.0	(33.0–73.0)	60.0	(44.0–69.0)	58.0	(33.0–73.0)	58.0	(34.0–73.0)

DVA = distance visual acuity; IQR = interquartile range; IVAN = Inhibition of VEGF in Age-related choroidal Neovascularisation; SE = study eye.

∗ Data missing for 1 patient (1 did not switch, 0 switched treatments, and 1 no injections).

† Data missing for 17 patients (−, 17 switched treatments).

‡ Data missing for 33 patients (0 did not switch, 0 switched treatments, 33 no injections).

‖ Switches include ranibizumab to aflibercept (n = 78) and bevacizumab to aflibercept (n = 3).

¶ Median predicted DVA from mixed effects model (Table 3) at median time to switch (2.7 years) is 61.2 for non-switchers.

### Change in Distance Visual Acuity per Year and Effect of Covariates on Change in Distance Visual Acuity

**Figure 4:**
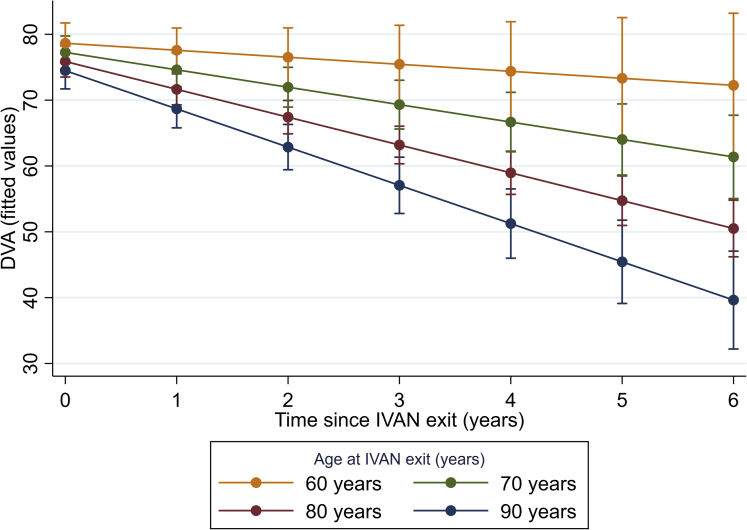
Change in distance visual acuity (DVA) by category of age at Inhibition of VEGF in Age-related choroidal Neovascularisation (IVAN) exit, DVA calculated from fitted values from the multivariable model.

Univariable and multivariable associations of time, patient-level characteristics and their interactions with rate of change in DVA from IVAN exit to the end of study eye monitoring are reported in Table 3. Main effects of age, BCVA at IVAN exit, and status of study eye remained significant in the multivariable analysis after adjusting for covariates. The model estimated the deterioration in DVA during study eye monitoring to be 4.3 letters per year (95% CI, 3.7–4.9) for a participant aged 80 years at IVAN exit. The only significant interaction was between age and time (*P* = 0.001), with DVA deteriorating faster in older participants and slower in younger participants (Fig 4); thus, the rate of deterioration was 3.0 letters per year for a 70-year-old and 5.6 letters per year for a 90-year-old.

**Table 3:** Effect Estimates from Univariable and Multivariable Models of Distance Visual Acuity in Study Eye during Study Eye Monitoring

Variable		Univariable		Multivariable		
		MD (95% CI)	P Value	MD (95% CI)	P Value	P Value for Interaction with Time
Time (per year), multivariable model				−4.3 (−4.9 to −3.7)	-	-
Time (per year)		−4.3 (−4.9 to −3.7)	-			
Age at IVAN exit (per 10 yrs), centered		−3.8 (−5.9 to −1.7)	-	−1.1 (−2.1 to −0.1)	-	-
Age at IVAN exit (per 10 yrs), centered × time		−1.2 (−2.1 to −0.4)	0.005	−1.3 (−2.1 to −0.5)‡	-	0.001
Time (per year)		−4.3 (−5.1 to −3.5)	-			
Gender (male)		1.0 (−2.2 to 4.2)	-	0.5 (−1.0 to 1.9)	0.529	0.916
Gender (male) × time		0.4 (−0.9 to 1.7)	0.579			
Time (per year)		−2.8 (−4.1 to −1.5)	-			
Index of multiple deprivation decile		0.4 (−0.2 to 0.9)	-	−0.2 (−0.4 to 0.1)	0.193	0.100§
Index of multiple deprivation decile × time		−0.2 (−0.4 to 0.0)	0.017			
Time (per year)		−4.2 (−4.9 to −3.4)	-			
BCVA at IVAN exit	≥68	*Ref.*	-	*Ref*.	<0.001	0.398
	53–67	−18.2 (−20.2 to −16.2)		−17.0 (−19.0 to −15.1)		
	38–52	−31.3 (−33.9 to −28.7)		−29.5 (−32.0 to −27.0)		
	≤37	−46.5 (−48.9 to −44.0)		−44.6 (−47.0 to −42.1)		
BCVA at IVAN exit × time	≥68	*Ref.*	0.326			
	53−67	−0.9 (−2.7 to 0.8)				
	38−52	1.4 (−0.8 to 3.6)				
	≤37	0.4 (−1.9 to 2.8)				
Time (per year)		−4.5 (−5.4 to −3.6)	-			
nAMD present in fellow eye		3.4 (0.2 to 6.5)	-	−0.9 (−2.6 to 0.7)	0.281	0.202
nAMD present in fellow eye × time		0.6 (−0.6 to 1.9)	0.324			
Time (per year)		−4.4 (−5.2 to −3.6)	-			
Study eye BCVA better than fellow eye at IVAN exit∗		12.6 (9.5 to 15.7)	-	3.9 (2.0 to 5.7)	<0.001	0.172
Study eye BCVA better than fellow eye at IVAN exit × time		0.6 (−0.7 to 1.9)	0.371			
Time (per year)		−4.2 (−4.8 to −3.6)	-			
Injection rate in study eye in previous year (per 3 injections)		−0.1 (−0.8 to 0.7)	-	−0.2 (−0.9 to 0.5)	0.549	0.056‖
Injection rate in study eye in previous year (per 3 injections) × time		0.2 (0.0 to 0.3)	0.056			
Time (per year)		−4.6 (−5.3 to −3.8)	-			
Proportion change in lesion size†		0.1 (−0.4 to 0.5)	-	0.0 (−0.1 to 0.1)	0.757	0.178
Proportion change in lesion size × time		0.1 (−0.1 to 0.4)	0.159			

BCVA = best-corrected visual acuity; CI = confidence interval; DVA = distance visual acuity; IMD = index of multiple deprivation; IVAN = Inhibition of VEGF in Age-related choroidal Neovascularisation; MD = mean difference; nAMD = neovascular age-related macular degeneration.

Injection rate in study eye in previous year (per 3 injections) × time = 0.2 (0.0–0.3)

∗ Study eye is defined as better than the fellow eye if study eye BCVA ≥5 letters greater than fellow eye BCVA at IVAN exit.

† Proportion change in lesion size between IVAN entry and IVAN exit (lesion size at IVAN exit/lesion size at IVAN entry).

‡ This interaction means that in an 80-year-old, the average rate of change in DVA in the study eye was −4.3 (95% CI, −4.9 to −3.7) letters per year but, for every 10-year increase in age, a further 1.3 letters per year were lost; thus, for a 90-year-old, the rate of change in DVA was −4.3 to 1.3 = −5.6 letters per year. See also Figure 4.

§ Interaction not included in final multivariable model. Effect estimates for IMD (main effect) and IMD by time interaction if included in the multivariable model are index of multiple deprivation decile = −0.1 (−0.4 to 0.1). Index of multiple deprivation decile × time = −0.2 (−0.4 to 0.1). Model fitted to n = 532 patients. Missing data imputed using multiple imputation methods. Table S10 gives complete case analysis. Age is centered at the average age (80 years). Because of small effect sizes for each 1-year increase in age, age is scaled per 10 years. Because of the small effect size for each increase in 1 injection, injection rate is scaled, and effect sizes reported are per 3 injections. Univariable models were fitted with time, covariate and time × covariate interaction only. Multivariable models were fitted with time, all covariates and time × age interaction.

‖ Interaction not included in final multivariable model. Effect estimates for injection rate (main effect) and injection rate by time interaction if included in the final multivariable model are injection rate in study eye in previous year (per 3 injections) = −0.5 (−1.2 to 0.3).

Other effects were constant from IVAN exit to the end of study eye monitoring. Relative to the contralateral eye, the average DVA of study eyes that were classified as “better seeing” at IVAN exit was estimated to be 3.9 letters better throughout compared with study eyes classified as “worse seeing” (Table 3). Injection rate in a preceding year did not influence the rate of change in vision in the subsequent year. Change in lesion size during the trial also had no effect on the rate of change in vision. The 3 sensitivity analyses shown in Tables S8 to S10 (available at www.aaojournal.org) showed similar results to that of the main model. Comparisons of DVA, mortality, and EQ-5D-5L found no differences between groups by original trial allocations (Figure S6 available at www.aaojournal.org).

## Discussion

This report describes the long-term functional outcomes in participants in the IVAN trial after release from protocol. Our main findings were as follows:

• DVA in the study eye deteriorated by more than 4 letters per year during the follow-up period. The rate of deterioration in DVA was faster for older participants and slower for younger ones.
• Injection rate did not influence the rate of change in DVA after taking account of covariates at IVAN exit.
• The majority of study eyes were commenced on ranibizumab and remained on this treatment throughout follow-up. Approximately one-fifth of study eyes switched treatment from ranibizumab to aflibercept.

Our findings confirm those of other long-term follow-up studies that anti-VEGF therapy has radically altered the natural history of untreated nAMD; overall, one-third of study eyes had a DVA of 68 letters or better 3 to 5 years after exiting the IVAN trial (i.e., 5–7 years after starting anti-VEGF treatment), maintaining a level of vision that allowed participants to continue driving. To estimate VA changes over time in patients receiving anti-VEGF therapy, investigators have attempted to obtain information on participants after exit from major clinical trials.^1^^,^^2^ However, the completeness of data has been a potential source of bias since the majority of trials and real world studies have reported data from less than 50% of their original cohorts.^1^, ^2^, ^3^, ^4^, ^5^, ^6^, ^7^^,^^13^

The most complete follow-up data to date are from the CATT trial, which measured BCVA 5 years after enrollment in 647 participants (70.7% of 914 invited to attend a research visit and 57.9% of those who had completed 2 years in the trial).^2^ Mean BCVA among this group declined by 11 letters over 3.5 years since trial exit, that is, 3.14 letters per year. Among those who attended for the research visit, the mean change in BCVA from IVAN exit to the research visit provides the best comparison; we found that this was −19.7 letters over 5.3 years (BCVA at IVAN exit = 71.5 letters [SD, 14.3] and BCVA at research visit = 51.8 letters [SD, 26.2]), that is, 3.72 letters per year. This estimate contrasts with the average reduction in DVA during study eye monitoring when the entire IVAN cohort including those who died during follow-up are included. We observed a reduction in DVA of 4.3 letters per year, based on the data that were available for 99% of the eligible cohort, highlighting the bias that can arise when only a selected subset of participants are considered. This type of bias has likely occurred in those reports in which the estimates of visual acuity decline are low. For example, Berg et al^3^ reported an overall loss of 2.1 ETDRS letters compared with baseline after 8 years of follow-up (and an improvement of 6.1 letters in the first year) using a treat and extend regimen, but less than 40% of the original cohort were included in the long-term VA outcomes. A recent study of 67 patients reported a mean VA after 7 years of follow-up of 65 letters (∼7 letters better than baseline) but only included 6.7% of patients who met their specific follow-up criteria.^14^

We chose to estimate the change in DVA during study eye monitoring (i.e., active surveillance), recognizing that participants might continue to attend usual care visits for surveillance and treatment of the fellow eye. We considered this to be the most appropriate period over which to estimate change, because it represents the time during which there was active management of the study eye to control neovascular lesion activity. Because we had limited information on reasons why study eye monitoring was stopped, we assumed that it ended on the date of the most recent study eye injection if that date was 1 year or more before the most recent usual care visit and injections were continuing to the fellow eye.

The multivariable analysis of DVA showed that the rate of deterioration during study eye monitoring was unaffected by the BCVA level at IVAN exit. This finding is concordant with previously published data on the outcomes of nAMD treated with anti VEGF therapies.^4^, ^5^, ^6^, ^7^ The multivariable analysis also revealed a strong interaction between age and time: DVA declined faster in older participants. Several studies^13^^,^^15^ have identified increasing age as an adverse predictor of visual outcome but have merely reported findings for dichotomized age groups, for example, comparing those aged 80 years and above with those younger than 80 years of age. Our finding is of high clinical relevance both for counseling and in the management of patients, acknowledging that age is likely a proxy for complex underlying comorbidity.

We also provide information on visit and injection rates by BCVA category at IVAN exit. We observed the lowest injection rates in study eyes were in the worst BCVA category at IVAN exit but accompanied by only a marginally lower visit rate compared with the other categories of BCVA, indicating that monitoring of the study eye was continuing despite the low level of vision. It was also notable that study eyes with BCVA better than 38 letters but less than 67 letters (the 2 middle categories of BCVA at IVAN exit) had similar visits and injection rates as the best BCVA category. Of note, injection rate did not significantly influence the rate of VA loss as shown in the multivariable model. This finding should be viewed with caution because it is contrary to other studies that report better VA outcomes with higher injection rates.^13^^,^^14^^,^^16^ In support of our observation, we have provided information on the distribution in injection rates across individuals (Figs 1 and 2 and Table S6 [available at www.aaojournal.org] in addition to the multivariable effects shown in detail in Table 3). There are 2 reasons why we believe our findings are robust. First, we calculated injection rate by each year of follow-up and analyzed its effect as a time-varying covariate, along with a sensitivity analysis. Second, we distinguished between a main effect of injection rate and the interaction of injection rate and time. Neither effect was statistically significant, and the point estimates were small (Table 3): −0.5 letters (main effect, at IVAN exit: eyes read 0.5 letters fewer per additional 3 injections) and 0.2 letters (interaction: eyes read 0.2 letters more per year per additional 3 injections).

We identified a group of study eyes in which the anti-VEGF agent that was commenced at IVAN exit was subsequently changed at some point during study eye monitoring. Compared with study eyes that were maintained on the same treatment throughout monitoring, switchers were younger, had marginally better BCVA at IVAN exit, were monitored for longer, and had similar DVA at the end of study eye monitoring to non-switchers. We did not attempt to compare the rate of change in DVA per year in study eyes or the number of treatments given in switchers with non-switchers because switch occurred at different times during study eye monitoring and the comparison would have been confounded by differences in the characteristics of the participants.

Although CATT reported a higher frequency of treatments compared with IVAN in their 5-year follow-up,^2^ their visual acuity outcomes were similar to ours. Westborg et al^16^ reported an average of 21 injections over a 7-year period from treatment initiation, and the gain in VA seen in initial years was lost with a mean change of −1 letter from baseline at the final follow-up. However, these represent findings from less than one-fifth of the original cohort of 322 patients, because data from 82% were not included.

The data on injection rates in the IVAN follow-up study are not easily compared with other published data for 2 main reasons. First, most long-term follow-up studies cite the injection numbers from treatment initiation that will reflect the high frequency of treatments in the early years after diagnosis. Second, in these studies, no data are available on participants lost to follow-up temporarily or permanently. Most studies have not reported data on reasons for ceasing treatment. Injection rate is also particularly susceptible to bias from attrition, because injections that are administered in those who are lost to follow-up cannot be counted. Unlike in previous studies, we calculated injection rates for almost all participants after release from trial protocol, including patients who had died during study eye monitoring.

### Strengths and Limitations

Key strengths of the study are the high proportion of the trial cohort for whom we collected information and the resistance to attrition during follow-up of the rate of change in DVA in usual care, our chosen visual function measure. We were able to analyze the rate of change in DVA because we extracted information on this measure at all usual care visits. We also showed that in a subset of participants who attended a research visit, DVA at the most recent monitoring visit agreed well with BCVA measured at the research visit. In this regard, our data differ markedly from real world studies^5^, ^6^, ^7^ and the longer-term follow-up outcomes reported on participants previously enrolled in RCTs of anti-VEGF therapies for nAMD.^1^, ^2^, ^3^ Given these strengths, we believe that our findings should provide a reference of the decline in visual acuity that ophthalmologists and health policy makers should expect to observe when treating nAMD with anti-VEGF drugs over 5 to 7 years after starting treatment.

There are 4 main limitations of the study. First, approximately one-half of the IVAN cohort were being actively monitored at the time data were extracted. However, we obtained data on 95% of IVAN participants from their usual care visits, and their trajectories were estimated over the period in which they received usual care; therefore, we believe that the estimates of decline in DVA that we show are likely to represent a truer picture than that reported previously.

Second, for the majority of the cohort we depended on DVAs measured in usual care. A large proportion of these were obtained on ETDRS charts, and by comparing DVA at the last monitoring visit with BCVA at the research visit among participants who attended a research visit within 60 days, we were able to show the validity of the DVA measurements (the average difference was only 1.8 letters). Third, we were unable to be sure when monitoring of the study eye ceased. This date was known when the fellow eye never developed nAMD during the IVAN trial or the follow-up study or when the fellow eye had a nAMD lesion on IVAN entry that was never treated. However, we did not know the date when study eye monitoring ceased if the fellow eye developed a nAMD lesion during the IVAN trial or follow-up study and continued to receive treatment after treatment to the study eye ceased. In these instances, we inferred the date on the basis of the time of cessation of treatment to the study eye if treatment continued in the fellow eye. Fourth, although the patterns of re-treatment that we observed suggest in the main that a pro re nata approach to patient management was used by the majority of sites, it is possible that a treat-and-extend posology was being used in some patients because this has been adopted widely in the United Kingdom and elsewhere in recent years.^14^ However, our data collection finished in August 2017, and we did not observe the emergence of any change in the patterns of re-treatment; therefore, we do not have evidence on how the treat-and-extend posology might have influenced visual outcomes. Nonetheless, images that were analyzed from the most recent patient visit suggested that the majority of eyes had dormant nAMD lesions as key markers of lesion activity consisting of intraretinal and subretinal fluid that were absent in the majority of eyes (data not reported or shown in this article).

In conclusion, 6 years after enrollment in IVAN, we have shown that approximately one-third of eyes with nAMD treated with anti-VEGF therapies can retain a level of vision that is sufficient for driving, but on average DVA deteriorates by approximately 4.3 ETDRS letters per year. Anti-VEGF injection rate was not an important factor in determining visual acuity outcome because injection rate per se did not influence the rate of DVA loss. Notable new findings include the characterization of the higher rate of visual acuity decline in older people. These data obtained during the IVAN follow-up study have important implications for healthcare providers in terms of planning and resource allocation and for clinicians in terms of patient counseling and treatment planning.

## References

[1] Rofagha S., Bhisitkul R.B., Boyer D.S. (2013). Seven-year outcomes in ranibizumab-treated patients in ANCHOR, MARINA, and HORIZON: a multicenter cohort study (SEVEN-UP). Ophthalmology.

[2] Maguire M.G., Martin D.F., Ying G.S. (2016). Five-year outcomes with anti-vascular endothelial growth factor treatment of neovascular age-related macular degeneration: the Comparison of Age-Related Macular Degeneration Treatments Trials. Ophthalmology.

[3] Berg K., Roald A.B., Navaratnam J., Bragadottir R. (2017). An 8-year follow-up of anti-vascular endothelial growth factor treatment with a treat-and-extend modality for neovascular age-related macular degeneration. Acta Ophthalmologica.

[4] Bakri S.J., Thorne J.E., Ho A.C. (2019). Safety and efficacy of anti-vascular endothelial growth factor therapies for neovascular age-related macular degeneration: a Report by the American Academy of Ophthalmology. Ophthalmology.

[5] Gillies M.C., Campain A., Barthelmes D. (2015). Long-term outcomes of treatment of neovascular age-related macular degeneration: data from an observational study. Ophthalmology.

[6] (2014). The neovascular age-related macular degeneration database: multicenter study of 92 976 ranibizumab injections: report 1: visual acuity. Ophthalmology.

[7] Johnston R.L., Lee A.Y., Buckle M. (2016). UK Age-Related Macular Degeneration Electronic Medical Record System (AMD EMR) Users Group Report IV: incidence of blindness and sight impairment in ranibizumab-treated patients. Ophthalmology.

[8] Chakravarthy U., Harding S.P., Rogers C.A. (2013). Alternative treatments to inhibit VEGF in age-related choroidal neovascularisation: 2-year findings of the IVAN randomised controlled trial. Lancet.

[9] Five year observational follow-up of the IVAN trial cohort: a study of function and morphology [12/04/2018]. https://www.journalslibrary.nihr.ac.uk/programmes/hta/0736501/#/.

[10] (1990). EuroQol--a new facility for the measurement of health-related quality of life. Health Policy (Amsterdam, Netherlands).

[11] Bland J.M., Altman D.G. (1994). Correlation, regression, and repeated data: Authors' reply. BMJ.

[12] National statistics 2015. https://www.gov.uk/government/statistics/english-indices-of-deprivation-2015.

[13] Nguyen C.L., Gillies M.C., Nguyen V. (2019). Characterization of poor visual outcomes of neovascular age-related macular degeneration treated with anti-vascular endothelial growth factor agents. Ophthalmology.

[14] Adrean S.D., Chaili S., Ramkumar H. (2018). Consistent long-term therapy of neovascular age-related macular degeneration managed by 50 or more anti-VEGF injections using a treat-extend-stop protocol. Ophthalmology.

[15] Chatziralli I., Nicholson L., Vrizidou E. (2016). Predictors of outcome in patients with neovascular age-related macular degeneration switched from ranibizumab to 8-weekly aflibercept. Ophthalmology.

[16] Westborg I., Granstam E., Rosso A. (2017). Treatment for neovascular age-related macular degeneration in Sweden: outcomes at seven years in the Swedish Macula Register. Acta Ophthalmologica.

