# Supplementary material for: Long-term Visual Outcomes after Release from Protocol in Patients who Participated in the Inhibition of VEGF in Age-related Choroidal Neovascularisation (IVAN) Trial
Source: Ophthalmology. 2020 Sep;127(9):1191–200. doi: 10.1016/j.ophtha.2020.03.020 (PMC7471837; doi:10.1016/j.ophtha.2020.03.020)
Supplement: Table S1 [file mmc1.docx]

**Table S1 Mapping of LogMAR, Snellen fraction and count fingers/hand movements/perception of light to ETDRS letters**

| **LogMAR** | **Snellen equiv.** | **No letters read** | **ETDRS letters read** |
| --- | --- | --- | --- |
| -0.3 | 6/3 |  | 98 |
| -0.2 | 6/4 |  | 93 |
| -0.1 | 6/5 |  | 88 |
| 0 | 6/6 |  | 83 |
| 0.1 | 6/7.5 |  | 78 |
| 0.2 | 6/9.5 |  | 73 |
| 0.3 | 6/12 |  | 68 |
| 0.4 | 6/15 |  | 63 |
| 0.5 | 6/19 |  | 58 |
| 0.6 | 6/24 |  | 53 |
| 0.7 | 6/30 |  | 48 |
| 0.8 | 6/38 |  | 43 |
| 0.9 | 6/48 |  | 38 |
| 1 | 6/60 |  | 33 |
| 1.1 | 6/75 |  | 28 |
| 1.2 | 6/96 |  | 23 |
| 1.3 | 6/120 |  | 18 |
| 1.4 | 6/150 |  | 13 |
| 1.5 | 6/190 |  | 8 |
| 1.6 | 6/240 |  | 3 |
|  |  | Count fingers | 0 |
|  |  | Hand movements | -15 |
|  |  | Perception of light | -30 |

**Abbreviations:** ETDRS=Early Treatment Diabetic Retinopathy Study
