# Supplementary material for: Long-term Visual Outcomes after Release from Protocol in Patients who Participated in the Inhibition of VEGF in Age-related Choroidal Neovascularisation (IVAN) Trial
Source: Ophthalmology. 2020 Sep;127(9):1191–200. doi: 10.1016/j.ophtha.2020.03.020 (PMC7471837; doi:10.1016/j.ophtha.2020.03.020)
Supplement: Table S2 [file mmc2.docx]

Table S2 IVAN follow-up cohort (n=532): demographics and medical history at IVAN entry and exit, by trial allocations

| **Characteristic** | **Randomised to ranibizumab (n=272)** | | **Randomised to bevacizumab (n=260)** | | **SMD** | **Randomised to continuous (n=269)** | | **Randomised to discontinuous (n=263)** | | **SMD** | **Overall (n=532)** | |
| --- | --- | --- | --- | --- | --- | --- | --- | --- | --- | --- | --- | --- |
|  | **mean** | **SD** | **mean** | **SD** | **Ranibizumab vs Bevacizumab** | **mean** | **SD** | **mean** | **SD** | **Continuous vs Discontinuous** | **mean** | **SD** |
| **Demography** |  |  |  |  |  |  |  |  |  |  |  |  |
| Age at IVAN exit visit, years | 79.3 | 7.6 | 79.6 | 7.2 | -0.05 | 79.5 | 8.0 | 79.4 | 6.7 | 0.01 | 79.4 | 7.4 |
| Male gender (n, %) | 111/272 | 40.8% | 101/260 | 38.8% | 0.04 | 109/269 | 40.5% | 103/263 | 39.2% | 0.03 | 212/532 | 39.8% |
| Blood pressure, mmHg |  |  |  |  |  |  |  |  |  |  |  |  |
| IVAN entry |  |  |  |  |  |  |  |  |  |  |  |  |
| Systolic | 141.4 | 19.0 | 143.4 | 19.4 | -0.11 | 143.5 | 19.8 | 141.2 | 18.5 | 0.12 | 142.4 | 19.2 |
| Diastolic | 76.5 | 10.4 | 77.5 | 10.0 | -0.10 | 77.6 | 10.3 | 76.3 | 10.1 | 0.12 | 77.0 | 10.2 |
| IVAN exit |  |  |  |  |  |  |  |  |  |  |  |  |
| Systolic ^a^ | 137.5 | 19.3 | 139.5 | 18.5 | -0.11 | 138.9 | 19.6 | 138.1 | 18.2 | 0.04 | 138.5 | 18.9 |
| Diastolic ^b^ | 74.0 | 10.0 | 74.6 | 10.4 | -0.06 | 75.0 | 10.0 | 73.6 | 10.4 | 0.13 | 74.3 | 10.2 |
| **Non-ocular past history (n, %)** |  |  |  |  |  |  |  |  |  |  |  |  |
| Angina | 29/272 | 10.7% | 43/260 | 16.5% | -0.17 | 38/269 | 14.1% | 34/263 | 12.9% | 0.04 | 72/532 | 13.5% |
| Dyspnoea |  |  |  |  |  |  |  |  |  |  |  |  |
| IVAN entry | 46/271 | 17.0% | 51/259 | 19.7% | -0.07 | 46/267 | 17.2% | 51/263 | 19.4% | -0.06 | 97/530 | 18.3% |
| IVAN exit | 49/271 | 18.1% | 57/259 | 22.0% | -0.10 | 52/268 | 19.4% | 54/262 | 20.6% | -0.03 | 106/530 | 20.0% |
| Myocardial Infarction | 19/272 | 7.0% | 18/260 | 6.9% | 0.00 | 22/269 | 8.2% | 15/263 | 5.7% | 0.10 | 37/532 | 7.0% |
| Transient ischemic attack | 18/254 | 7.1% | 8/249 | 3.2% | 0.18 | 14/252 | 5.6% | 12/251 | 4.8% | 0.03 | 26/503 | 5.2% |
| Stroke | 3/272 | 1.1% | 5/260 | 1.9% | -0.07 | 3/269 | 1.1% | 5/263 | 1.9% | -0.06 | 8/532 | 1.5% |
| DVT/PE | 13/271 | 4.8% | 17/260 | 6.5% | -0.08 | 14/269 | 5.2% | 16/262 | 6.1% | -0.04 | 30/531 | 5.6% |
| Current or past smoker | 173/268 | 64.6% | 158/260 | 60.8% | 0.08 | 170/266 | 63.9% | 161/262 | 61.5% | 0.05 | 331/528 | 62.7% |

^a^ Data missing for 8 patients (4 Ranibizumab, 4 Bevacizumab | 6 continuous, 2 discontinuous)

^b^ Data missing for 8 patients (4 Ranibizumab, 4 Bevacizumab | 6 continuous, 2 discontinuous)

**Abbreviations:** DVT=Deep vein thrombosis, PE=Pulmonary embolism, SD=Standard deviation, SMD=Standardised mean difference
