# Supplementary material for: Long-term Visual Outcomes after Release from Protocol in Patients who Participated in the Inhibition of VEGF in Age-related Choroidal Neovascularisation (IVAN) Trial
Source: Ophthalmology. 2020 Sep;127(9):1191–200. doi: 10.1016/j.ophtha.2020.03.020 (PMC7471837; doi:10.1016/j.ophtha.2020.03.020)
Supplement: Table S3 [file mmc3.docx]

Table S3 Non-ocular and ocular history after release from protocol in IVAN trial (research visit attenders only)

| **Event** |  | **Randomised to ranibizumab (n=105)** | | **Randomised to bevacizumab (n=94)** | | **Randomised to continuous (n=101)** | | **Randomised to discontinuous (n=98)** | | **Overall (n=199)** | |
| --- | --- | --- | --- | --- | --- | --- | --- | --- | --- | --- | --- |
|  |  | **n** | **%** | **n** | **%** | **n** | **%** | **n** | **%** | **n** | **%** |
| **Non-ocular history** |  |  |  |  |  |  |  |  |  |  |  |
| Angina |  | 2/105 | 1.9% | 1/94 | 1.1% | 1/101 | 1.0% | 2/98 | 2.0% | 3/199 | 1.5% |
| Dyspnoea |  | 12/105 | 11.4% | 13/94 | 13.8% | 11/101 | 10.9% | 14/98 | 14.3% | 25/199 | 12.6% |
| Claudication |  | 2/105 | 1.9% | 1/94 | 1.1% | 1/101 | 1.0% | 2/98 | 2.0% | 3/199 | 1.5% |
| Transient ischemic attack |  | 3/105 | 2.9% | 3/94 | 3.2% | 2/101 | 2.0% | 4/98 | 4.1% | 6/199 | 3.0% |
| Stroke |  | 2/105 | 1.9% | 1/94 | 1.1% | 1/101 | 1.0% | 2/98 | 2.0% | 3/199 | 1.5% |
| DVT/PE |  | 1/105 | 1.0% | 0/94 | 0.0% | 0/101 | 0.0% | 1/98 | 1.0% | 1/199 | 0.5% |
| Diabetes |  | 6/105 | 5.7% | 3/94 | 3.2% | 4/101 | 4.0% | 5/98 | 5.1% | 9/199 | 4.5% |
| **Ocular history** |  |  |  |  |  |  |  |  |  |  |  |
| Cataract surgery | Study eye | 8/50 | 16.0% | 11/48 | 22.9% | 11/53 | 20.8% | 8/45 | 17.8% | 19/98 | 19.4% |
|  | Fellow eye | 6/50 | 12.0% | 10/48 | 20.8% | 9/53 | 17.0% | 7/45 | 15.6% | 16/98 | 16.3% |
| Diabetic eye disease | Study eye | 0/105 | 0.0% | 1/94 | 1.1% | 1/101 | 1.0% | 0/98 | 0.0% | 1/199 | 0.5% |
|  | Fellow eye | 0/105 | 0.0% | 1/94 | 1.1% | 1/101 | 1.0% | 0/98 | 0.0% | 1/199 | 0.5% |
| Glaucoma | Study eye | 5/105 | 4.8% | 2/94 | 2.1% | 4/101 | 4.0% | 3/98 | 3.1% | 7/199 | 3.5% |
|  | Fellow eye | 3/105 | 2.9% | 2/94 | 2.1% | 2/101 | 2.0% | 3/98 | 3.1% | 5/199 | 2.5% |
| nAMD | Fellow eye | 21/104 | 20.2% | 21/93 | 22.6% | 21/101 | 20.8% | 21/96 | 21.9% | 42/197 | 21.3% |
| Episode of endophthalmitis | Study eye | 0/105 | 0.0% | 0/94 | 0.0% | 0/101 | 0.0% | 0/98 | 0.0% | 0/199 | 0.0% |
|  | Fellow eye | 0/105 | 0.0% | 1/94 | 1.1% | 0/101 | 0.0% | 1/98 | 1.0% | 1/199 | 0.5% |
| Vascular occlusion | Study eye | 1/105 | 1.0% | 0/94 | 0.0% | 0/101 | 0.0% | 1/98 | 1.0% | 1/199 | 0.5% |
|  | Fellow eye | 1/105 | 1.0% | 1/94 | 1.1% | 1/101 | 1.0% | 1/98 | 1.0% | 2/199 | 1.0% |

**Abbreviations:** DVT=Deep vein thrombosis, PE=Pulmonary embolism, nAMD= neovascular age-related macular degeneration
