# Supplementary material for: Long-term Visual Outcomes after Release from Protocol in Patients who Participated in the Inhibition of VEGF in Age-related Choroidal Neovascularisation (IVAN) Trial
Source: Ophthalmology. 2020 Sep;127(9):1191–200. doi: 10.1016/j.ophtha.2020.03.020 (PMC7471837; doi:10.1016/j.ophtha.2020.03.020)
Supplement: Table S4 [file mmc4.docx]

Table S4 Certificate of visual impairment (CVI) of participants in the IVAN trial after release from protocol

| **CVI** |  | **Randomised to ranibizumab (n=272)** | | **Randomised to bevacizumab (n=260)** | | **Randomised to continuous (n=269)** | | **Randomised to discontinuous (n=263)** | | **Overall (n=532)** | |
| --- | --- | --- | --- | --- | --- | --- | --- | --- | --- | --- | --- |
|  |  | **n** | **%** | **n** | **%** | **n** | **%** | **n** | **%** | **n** | **%** |
| CVI issued |  | 41/272 | 15.1% | 30/260 | 11.5% | 34/269 | 12.6% | 37/263 | 14.1% | 71/532 | 13.3% |
| Level | Severely sight impaired (blind) | 12/41 | 29.3% | 7/30 | 23.3% | 7/34 | 20.6% | 12/37 | 32.4% | 19/71 | 26.8% |
|  | Sight impaired (partially sighted) | 29/41 | 70.7% | 23/30 | 76.7% | 27/34 | 79.4% | 25/37 | 67.6% | 52/71 | 73.2% |
| CVI re-issued |  | 3/272 | 1.1% | 2/260 | 0.8% | 4/269 | 1.5% | 1/263 | 0.4% | 5/532 | 0.9% |
| Level | Severely sight impaired (blind) | 3/3 | 100.0% | 2/2 | 100.0% | 4/4 | 100.0% | 1/1 | 100.0% | 5/5 | 100.0% |
|  | Sight impaired (partially sighted) | 0/3 | 0.0% | 0/2 | 0.0% | 0/4 | 0.0% | 0/1 | 0.0% | 0/5 | 0.0% |

**Abbreviations:** CVI=Certificate of visual impairment
