# Supplementary material for: Long-term Visual Outcomes after Release from Protocol in Patients who Participated in the Inhibition of VEGF in Age-related Choroidal Neovascularisation (IVAN) Trial
Source: Ophthalmology. 2020 Sep;127(9):1191–200. doi: 10.1016/j.ophtha.2020.03.020 (PMC7471837; doi:10.1016/j.ophtha.2020.03.020)
Supplement: Table S5 [file mmc5.docx]

Table S5 Duration of study eye monitoring, number of visits and number of injections

| **Outcome** | **Overall (n=532)** | |  |
| --- | --- | --- | --- |
|  | **n** | **%** | |
| Any usual care appointments since IVAN exit |  |  | |
| None | 26/532 | 4.9% | |
| At least one | 506/532 | 95.1% | |
| Duration of study eye monitoring (years, Median IQR) |  |  | |
| Since IVAN entry | 5.2 | (3.3, 6.7) | |
| Since IVAN exit visit | 3.3 | (1.3, 4.7) | |
| Total number of visits (Median, IQR)* |  |  | |
| Since IVAN entry | 43.5 | (30.0, 67.0) | |
| Since IVAN exit visit | 19.0 | (6.0, 42.5) | |
| Total number of injections (Median, IQR)* |  |  | |
| Since IVAN entry | 24.0 | (18.5, 36.0) | |
| Since IVAN exit visit | 4.0 | (0.0, 17.0) | |

* During period of study eye monitoring

**Notes:** Pairs of injections that were recorded within 25 days each other were assumed to be recording errors and merged with the intervention assigned to later of the two dates (n=57 pairs).

**Abbreviations:** IQR= Interquartile range
