# Supplementary material for: Long-term Visual Outcomes after Release from Protocol in Patients who Participated in the Inhibition of VEGF in Age-related Choroidal Neovascularisation (IVAN) Trial
Source: Ophthalmology. 2020 Sep;127(9):1191–200. doi: 10.1016/j.ophtha.2020.03.020 (PMC7471837; doi:10.1016/j.ophtha.2020.03.020)
Supplement: Table S6 [file mmc6.docx]

Table S6 Visit and injection rate in study eye after release from protocol by year of follow up

| **BCVA at exit ^b^** | | **Year 1 (n=499)** | | **Year 2 (n=417)** | | **Year 3 (n=325)** | | **Year 4 (n=260)** | | **Year 5 (n=206)** | | **Year 6 (n=105)** | | **Year 7 (n=27)** | |
| --- | --- | --- | --- | --- | --- | --- | --- | --- | --- | --- | --- | --- | --- | --- | --- |
|  |  | **median** | **IQR** | **median** | **IQR** | **median** | **IQR** | **median** | **IQR** | **median** | **IQR** | **median** | **IQR** | **median** | IQR |
| **≤37** | **Monitored (n)** | 53 |  | 35 |  | 18 |  | 12 |  | 7 |  | 2 |  |  |  |
|  | Visit rate | 6.2 | (4.0, 9.0) | 6.4 | (3.7, 9.0) | 4.5 | (2.2, 10.0) | 5.5 | (3.0, 8.5) | 7.2 | (5.0, 8.7) | 7.2 | (5.2, 9.2) |  |  |
|  | Injection rate | 0.0 | (0.0, 1.0) | 0.0 | (0.0, 0.0) | 0.0 | (0.0, 0.0) | 0.0 | (0.0, 3.0) | 0.0 | (0.0, 6.6) | 0.0 | (0.0, 0.0) |  |  |
| **38-52** | **Monitored (n)** | 45 |  | 38 |  | 32 |  | 26 |  | 23 |  | 14 |  | 2 |  |
|  | Visit rate | 8.0 | (6.1, 10.1) | 9.5 | (7.0, 12.0) | 7.8 | (5.4, 12.0) | 8.0 | (4.0, 11.0) | 8.5 | (5.0, 11.0) | 11.2 | (6.4, 13.1) | 6.9 | (6.7, 7.0) |
|  | Injection rate | 2.0 | (0.0, 5.0) | 2.0 | (0.0, 4.8) | 2.0 | (0.0, 5.0) | 2.5 | (0.0, 5.0) | 1.0 | (0.0, 5.0) | 0.0 | (0.0, 3.0) | 3.0 | (0.0, 6.0) |
| **53-67** | **Monitored (n)** | 87 |  | 69 |  | 50 |  | 35 |  | 24 |  | 17 |  | 6 |  |
|  | Visit rate | 8.0 | (5.0, 11.0) | 8.1 | (6.0, 11.0) | 8.0 | (4.9, 10.0) | 8.0 | (6.0, 10.0) | 8.0 | (5.8, 9.5) | 7.0 | (6.3, 9.1) | 8.2 | (5.6, 9.5) |
|  | Injection rate | 2.0 | (0.0, 6.0) | 3.0 | (0.0, 5.3) | 2.0 | (0.0, 5.0) | 3.0 | (0.0, 5.4) | 3.0 | (0.0, 6.0) | 2.0 | (0.0, 4.2) | 5.4 | (0.0, 8.1) |
| **≥68** | **Monitored (n)** | 310 |  | 261 |  | 218 |  | 184 |  | 145 |  | 68 |  | 11 |  |
|  | Visit rate | 9.0 | (6.0, 11.0) | 9.0 | (7.0, 12.0) | 9.0 | (6.9, 12.0) | 9.0 | (6.1, 11.4) | 9.0 | (6.9, 12.0) | 9.3 | (7.9, 12.1) | 7.5 | (5.3, 11.8) |
|  | Injection rate | 3.0 | (0.0, 5.0) | 3.0 | (0.0, 6.0) | 4.0 | (0.0, 6.0) | 4.0 | (0.0, 6.2) | 4.3 | (0.0, 6.9) | 5.2 | (2.0, 7.4) | 5.3 | (1.3, 7.5) |
| **Overall** | **Monitored ^a^ (n)** | 496 |  | 404 |  | 319 |  | 258 |  | 200 |  | 101 |  | 19 |  |
|  | Visit rate | 9.0 | (6.0, 11.0) | 9.0 | (6.0, 12.0) | 8.0 | (6.0, 11.2) | 9.0 | (6.0, 11.0) | 8.6 | (6.1, 11.5) | 9.1 | (7.0, 12.0) | 7.5 | (5.6, 11.2) |
|  | Injection rate | 2.0 | (0.0, 5.0) | 3.0 | (0.0, 6.0) | 3.0 | (0.0, 6.0) | 4.0 | (0.0, 6.0) | 4.0 | (0.0, 6.6) | 4.0 | (0.0, 7.0) | 5.3 | (0.0, 7.5) |

**Note:**

Summaries are based on period of study eye monitoring. 33 patients study eye monitoring did not continue into extended follow-up (26 had no appointments since IVAN exit and 7 patients had no VA readings since IVAN exit), hence n=532-33=499 in year 1.

Study eye monitoring ended in: Year 1 n=82, Year 2 n=92, Year 3 n=65, Year 4 n=54, Year 5 n=101, Year 6 n=78, Year 7 n=26.

^a^ Patients monitored for less than 28 days during year excluded from that years summary: Year 1 n=3, Year 2 n=13, Year 3 n=6, Year 4 n= 2, Year 5 n= 6, Year 6 n=4, Year 7 n=8)

^b^ BCVA at IVAN exit missing for n=1 patient

Pairs of injections that were recorded within 25 days each other were assumed to be recording errors and merged with the intervention assigned to later of the two dates (n=57 pairs).

**Abbreviations:** BCVA=Best corrected visual acuity, IQR=Interquartile range
