# Supplementary material for: Long-term Visual Outcomes after Release from Protocol in Patients who Participated in the Inhibition of VEGF in Age-related Choroidal Neovascularisation (IVAN) Trial
Source: Ophthalmology. 2020 Sep;127(9):1191–200. doi: 10.1016/j.ophtha.2020.03.020 (PMC7471837; doi:10.1016/j.ophtha.2020.03.020)
Supplement: Table S7 [file mmc7.docx]

Table S7 Patient demography and past history, by treatment switch status

| **Characteristic** |  | **Did not switch * (n=277)** | | **Switched treatments *  (n=81)** | | **No treatments post IVAN exit  (n=174)** | | **Overall (n=532)** | |
| --- | --- | --- | --- | --- | --- | --- | --- | --- | --- |
|  |  | **Mean** | **SD** | **Mean** | **SD** | **Mean** | **SD** | **Mean** | **SD** |
| **Demography** |  |  |  |  |  |  |  |  |  |
| Age at IVAN exit visit, years |  | 79.4 | 7.2 | 77.4 | 7.4 | 80.5 | 7.5 | 79.4 | 7.4 |
| Male gender (n, %) |  | 106/277 | 38.3% | 38/81 | 46.9% | 68/174 | 39.1% | 212/532 | 39.8% |
| IMD decile (median, IQR) ^a^ |  | 6.0 | (3.0, 9.0) | 6.0 | (2.0, 9.0) | 6.0 | (3.0, 9.0) | 6.0 | (3.0, 9.0) |
| Blood pressure, mmHg |  |  |  |  |  |  |  |  |  |
| IVAN entry |  |  |  |  |  |  |  |  |  |
| Systolic |  | 141.2 | 18.6 | 141.5 | 19.0 | 144.8 | 20.0 | 142.4 | 19.2 |
| Diastolic |  | 76.7 | 10.3 | 77.2 | 9.6 | 77.2 | 10.4 | 77.0 | 10.2 |
| IVAN exit |  |  |  |  |  |  |  |  |  |
| Systolic ^b^ |  | 137.8 | 19.2 | 139.3 | 16.1 | 139.2 | 19.6 | 138.5 | 18.9 |
| Diastolic ^c^ |  | 74.1 | 10.2 | 75.5 | 8.7 | 74.1 | 10.8 | 74.3 | 10.2 |
| **Non-ocular past history (n, %)** |  |  |  |  |  |  |  |  |  |
| Angina |  | 32/277 | 11.6% | 8/81 | 9.9% | 32/174 | 18.4% | 72/532 | 13.5% |
| Dyspnoea |  |  |  |  |  |  |  |  |  |
| IVAN entry |  | 52/276 | 18.8% | 9/80 | 11.3% | 36/174 | 20.7% | 97/530 | 18.3% |
| IVAN exit |  | 52/277 | 18.8% | 14/81 | 17.3% | 40/172 | 23.3% | 106/530 | 20.0% |
| Myocardial Infarction |  | 22/277 | 7.9% | 4/81 | 4.9% | 11/174 | 6.3% | 37/532 | 7.0% |
| Transient ischemic attack |  | 17/267 | 6.4% | 3/73 | 4.1% | 6/163 | 3.7% | 26/503 | 5.2% |
| Stroke |  | 4/277 | 1.4% | 1/81 | 1.2% | 3/174 | 1.7% | 8/532 | 1.5% |
| DVT/PE |  | 15/276 | 5.4% | 4/81 | 4.9% | 11/174 | 6.3% | 30/531 | 5.6% |
| Current or past smoker |  | 170/274 | 62.0% | 58/81 | 71.6% | 103/173 | 59.5% | 331/528 | 62.7% |

**Notes:**

* Switches include: Ranibizumab to Aflibercept (n=78), Bevacizumab to Aflibercept (n=3)

^a^ Data missing for 37 patients (17 did not switch, 6 switched treatments, 14 no injections post IVAN)

^b^ Data missing for 8 patients (2 did not switch, 2 switched treatments, 4 no injections post IVAN)

^c^ Data missing for 8 patients (2 did not switch, 2 switched treatments, 4 no injections post IVAN)

**Abbreviations:** DVT=Deep vein thrombosis, PE=Pulmonary embolism, SD=Standard deviation, SMD=Standardised mean difference
