# Supplementary material for: Long-term Visual Outcomes after Release from Protocol in Patients who Participated in the Inhibition of VEGF in Age-related Choroidal Neovascularisation (IVAN) Trial
Source: Ophthalmology. 2020 Sep;127(9):1191–200. doi: 10.1016/j.ophtha.2020.03.020 (PMC7471837; doi:10.1016/j.ophtha.2020.03.020)
Supplement: Table S8 [file mmc8.docx]

Table S8 Sensitivity analysis after removal of visits during the first year after IVAN exit Effect estimates for covariates from the multivariable model of distance visual acuity (DVA)

| **Variable** |  | **MD (95% CI)** | **P value** | **P -value for interaction with time** |
| --- | --- | --- | --- | --- |
| Time (per year) |  | -3.2 (-3.8, -2.5) | - | *-* |
| Age at IVAN exit (per 10 years), centred |  | -1.8 (-3.8, 0.2) | - | - |
| Age (per 10 years), centred x time |  | -0.8 (-1.7, 0.1) | *-* | 0.069 |
| Gender (male) |  | 1.3 (-1.3, 3.8) | 0.329 | 0.895 |
| Index of multiple deprivation decile |  | -0.7 (-1.1, -0.3) | 0.001 | 0.881 |
| Best corrected visual acuity at IVAN exit | ≥68 | *Ref.* | <0.001 | 0.360 |
|  | 53-67 | -19.4 (-22.7, -16.1) |  |  |
|  | 38-52 | -29.9 (-34.3, -25.6) |  |  |
|  | ≤37 | -42.8 (-47.7, -38.0) |  |  |
| nAMD present in fellow eye |  | -2.5 (-5.4, 0.4) | 0.088 | 0.245 |
| Study eye BCVA better than fellow eye at IVAN exit ^a^ |  | 7.0 (3.8, 10.2) | <0.001 | 0.678 |
| Injection rate in study eye in previous year (per 3 injections) |  | 0.1 (-0.5, 0.7) | 0.832 | 0.550 |
| Proportion change in lesion size ^b^ |  | 0.1 (-0.1, 0.3) | 0.492 | 0.361 |

^a^ Study eye is defined as better than the fellow eye if study eye BCVA ≥5 letters greater than fellow eye BCVA at IVAN exit

^b^ Proportion change in lesion size between IVAN entry and IVAN exit (lesion size at IVAN exit/lesion size at IVAN entry)

**Abbreviations:** nAMD= neovascular age-related macular degeneration, BCVA=Best corrected visual acuity, CI=Confidence interval, MD=Mean difference
