# Supplementary material for: Long-term Visual Outcomes after Release from Protocol in Patients who Participated in the Inhibition of VEGF in Age-related Choroidal Neovascularisation (IVAN) Trial
Source: Ophthalmology. 2020 Sep;127(9):1191–200. doi: 10.1016/j.ophtha.2020.03.020 (PMC7471837; doi:10.1016/j.ophtha.2020.03.020)
Supplement: Table S9 [file mmc9.docx]

Table S9 Sensitivity analysis retaining visits that occurred after study eye monitoring had ended. Effect estimates of covariates from multivariable model of distance visual acuity (DVA).

| **Variable** |  | **MD (95% CI)** | **P value** | **P -value for interaction with time** |
| --- | --- | --- | --- | --- |
| Time (per year) |  | -4.1 (-4.7, -3.6) | - | *-* |
| Age at IVAN exit (per 10 years), centred |  | -1.3 (-2.3, 0.2) | - | - |
| Age (per 10 years), centred x time |  | -0.9 (-1.6, -0.1) | *-* | 0.019 |
| Gender (male) |  | 0.6 (-0.9, 2.0) | 0.460 | 0.689 |
| Index of multiple deprivation decile |  | -0.2 (-0.4, 0.1) | 0.192 | 0.079 |
| Best corrected visual acuity at IVAN exit | ≥68 | *Ref.* | <0.001 | 0.172 |
|  | 53-67 | -17.0 (-19.0, -15.1) |  |  |
|  | 38-52 | -29.7 (-32.3, -27.2) |  |  |
|  | ≤37 | -44.7 (-47.2, -42.1) |  |  |
| nAMD present in fellow eye |  | -1.2 (-2.9, 0.5) | 0.167 | 0.141 |
| Study eye BCVA better than fellow eye at IVAN exit ^a^ |  | 3.9 (2.1, 5.9) | <0.001 | 0.194 |
| Injection rate in study eye in previous year (per 3 injections) |  | 0.0 (-0.7, 0.6) | 0.896 | 0.912 |
| Proportion change in lesion size ^b^ |  | 0.0 (-0.1, 0.1) | 0.834 | 0.242 |

^a^ Study eye is defined as better than the fellow eye if study eye BCVA ≥5 letters greater than fellow eye BCVA at IVAN exit

^b^ Proportion change in lesion size between IVAN entry and IVAN exit (lesion size at IVAN exit/lesion size at IVAN entry)

**Abbreviations:** nAMD= neovascular age-related macular degeneration, BCVA=Best corrected visual acuity, CI=Confidence interval, MD=Mean difference
