# Supplementary material for: Long-term Visual Outcomes after Release from Protocol in Patients who Participated in the Inhibition of VEGF in Age-related Choroidal Neovascularisation (IVAN) Trial
Source: Ophthalmology. 2020 Sep;127(9):1191–200. doi: 10.1016/j.ophtha.2020.03.020 (PMC7471837; doi:10.1016/j.ophtha.2020.03.020)
Supplement: Table S10 [file mmc10.docx]

Table S10 Sensitivity analysis excluding eyes with missing covariates. Effect estimates of covariates from multivariable model of distance visual acuity (DVA).

| **Variable** |  | **MD (95% CI)** | **P value** | **P -value for interaction with time** |
| --- | --- | --- | --- | --- |
| Time (per year) |  | -4.2 (-4.9, -3.6) | - | *-* |
| Age at IVAN exit (per 10 years), centred |  | -1.4 (-2.6, 0.2) | - | - |
| Age (per 10 years), centred x time |  | -1.6 (-2.5, -0.7) | *-* | <0.001 |
| Gender (male) |  | 0.2 (-1.4, 1.9) | 0.776 | 0.601 |
| Index of multiple deprivation decile |  | -0.2 (-0.5, 0.0) | 0.098 | 0.271 |
| Best corrected visual acuity at IVAN exit | ≥68 | *Ref.* | <0.001 | 0.180 |
|  | 53-67 | -17.1 (-19.4, -14.8) |  |  |
|  | 38-52 | -29.3 (-32.0, -26.6) |  |  |
|  | ≤37 | -44.0 (-46.8, -41.2) |  |  |
| nAMD present in fellow eye |  | -1.3 (-3.1, 0.5) | 0.164 | 0.224 |
| Study eye BCVA better than fellow eye at IVAN exit ^a^ |  | 4.0 (2.0, 5.9) | <0.001 | 0.512 |
| Injection rate in study eye in previous year (per 3 injections) |  | -0.2 (-1.0, 0.6) | 0.635 | 0.104 |
| Proportion change in lesion size ^b^ |  | 0.0 (-0.4, 0.3) | 0.903 | 0.638 |

^a^ Study eye is defined as better than the fellow eye if study eye BCVA ≥5 letters greater than fellow eye BCVA at IVAN exit

^b^ Proportion change in lesion size between IVAN entry and IVAN exit (lesion size at IVAN exit/lesion size at IVAN entry)

**Notes:** Model fitted to patients with all data available for regression models. n=532 – 124 ^a^ – 5^b^ = 408. Exclusions: ^a^ Data missing for 124 patients: IMD decile (n=37), BCVA at IVAN exit (n=2), nAMD in FE (n=2), study/fellow eye better at IVAN exit (n=26), proportion change in lesion size (n=83); ^b^ 5 outliers removed after assessing model fit

Age is centred at the average age (80 years). Due to small effect sizes for each 1-year increase in age, age is scaled per 10 years.

Due to the small effect size for each increase in 1 injection, injection rate is scaled, and effect sizes reported are per 3 injections.

**Abbreviations:** DVA= Distance visual acuity, IMD= Index of multiple deprivation, nAMD= neovascular age-related macular degeneration, BCVA=Best corrected visual acuity, CI=Confidence interval
