# Supplementary material for: Long-term Visual Outcomes after Release from Protocol in Patients who Participated in the Inhibition of VEGF in Age-related Choroidal Neovascularisation (IVAN) Trial
Source: Ophthalmology. 2020 Sep;127(9):1191–200. doi: 10.1016/j.ophtha.2020.03.020 (PMC7471837; doi:10.1016/j.ophtha.2020.03.020)
Supplement: Figure S1 [file mmc11.docx]

Figure S1 Flow of participants; see text for definition of usual care visits

IVAN trial patients (n=23 sites, n=610 patients)

Participating sites: died/withdrawn from IVAN (n=66 patients)

Alive at the end of IVAN and did not withdraw from IVAN

(n=20 sites, 2 had merged, n=537 patients)

Deceased,
usual care data (n=124, 23%)

(62 ranibizumab, 62 bevacizumab:
65 continuous, 59 discontinuous)

Alive (n=413)

Attended a research visit,
usual care data (n=199, 37%)

(105 ranibizumab, 94 bevacizumab;
101 continuous, 98 discontinuous)

Did not attend a research visit, usual care data (n=209, 39%)

(105 ranibizumab, 104 bevacizumab;
103 continuous, 106 discontinuous)

Withdrawn from follow up (n=5, 1%)

Site not participating (n=2 sites; n=7 patients)

Alive and did not withdraw (n=408)

No usual care visits since IVAN exit
(n=3)

At least one usual care visit since IVAN exit

(n=196)

No usual care visits since IVAN exit
(n=11)

No usual care visits since IVAN exit
(n=12)

At least one usual care visit since IVAN exit (n=198)

At least one usual care visit since IVAN exit (n=112)

**Abbreviations:** IQR= Interquartile range
