# Supplementary material for: Long-term Visual Outcomes after Release from Protocol in Patients who Participated in the Inhibition of VEGF in Age-related Choroidal Neovascularisation (IVAN) Trial
Source: Ophthalmology. 2020 Sep;127(9):1191–200. doi: 10.1016/j.ophtha.2020.03.020 (PMC7471837; doi:10.1016/j.ophtha.2020.03.020)
Supplement: Figure S2 [file mmc12.docx]

Figure S2 Kaplan-Meier curve of time to end of study eye monitoring, by BCVA category at IVAN exit

**Note:** BCVA at IVAN exit missing for n=2 patients.

**Abbreviations:** BCVA=Best corrected visual acuity
