# Supplementary material for: Long-term Visual Outcomes after Release from Protocol in Patients who Participated in the Inhibition of VEGF in Age-related Choroidal Neovascularisation (IVAN) Trial
Source: Ophthalmology. 2020 Sep;127(9):1191–200. doi: 10.1016/j.ophtha.2020.03.020 (PMC7471837; doi:10.1016/j.ophtha.2020.03.020)
Supplement: Figure S3 [file mmc13.docx]

Figure S3 Change in distance visual acuity (DVA) in study eyes from IVAN exit visit by BCVA category at IVAN exit

**Notes:** n=number of patients with at least one VA reading in that year.

**Abbreviations:** DVA= Distance visual acuity, BCVA=Best corrected visual acuity
