# Supplementary material for: Long-term Visual Outcomes after Release from Protocol in Patients who Participated in the Inhibition of VEGF in Age-related Choroidal Neovascularisation (IVAN) Trial
Source: Ophthalmology. 2020 Sep;127(9):1191–200. doi: 10.1016/j.ophtha.2020.03.020 (PMC7471837; doi:10.1016/j.ophtha.2020.03.020)
Supplement: Figure S4 [file mmc14.docx]

Figure S4 Bland Altman plot of BCVA at research visit and DVA at most recent usual care visit in those who attended the research visit

**Note:** Restricted to visits within 60 days of each other (n=95). Mean difference = -1.83 (95% CI -4.25, 0.59)

**Abbreviations:** DVA= Distance visual acuity, BCVA=Best corrected visual acuity
