# Supplementary material for: Long-term Visual Outcomes after Release from Protocol in Patients who Participated in the Inhibition of VEGF in Age-related Choroidal Neovascularisation (IVAN) Trial
Source: Ophthalmology. 2020 Sep;127(9):1191–200. doi: 10.1016/j.ophtha.2020.03.020 (PMC7471837; doi:10.1016/j.ophtha.2020.03.020)
Supplement: Figure S6 [file mmc16.docx]

Figure S6 Distance visual acuity (DVA) in study eye, survival time and generic health status (EQ-5D-5L), by original trial allocations


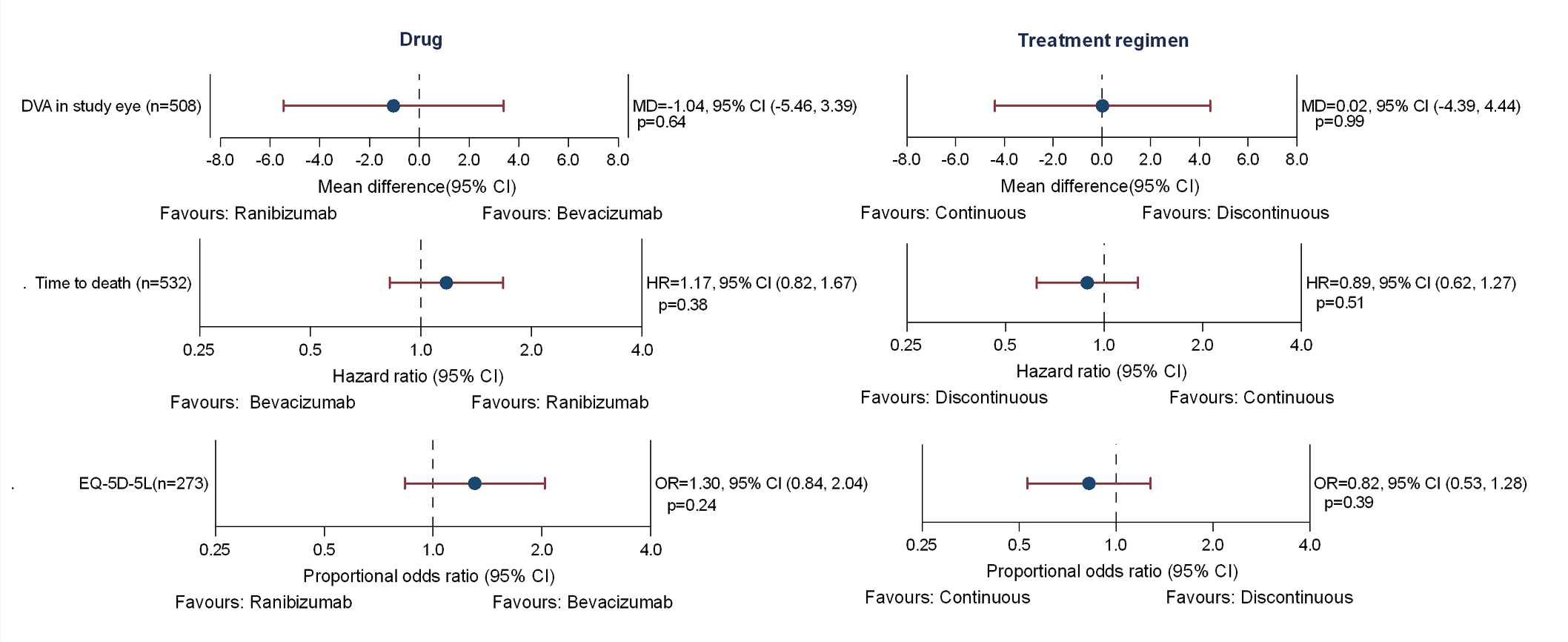


**Notes:** All estimates are adjusted for centre size.

DVA in study eye at end of extended follow-up (most recent measurement, including research visit for attenders) model fitted to IVAN follow up population, excluding 24 patients who do not have a DVA reading since IVAN exit (n=532-24 = 508).

Time to death model fitted to IVAN follow up population (n=532)

EQ-5D-5L model fitted to patients who attended and patients who did not attend but who opted to complete the questionnaire (n=285), excluding 12 patients who did not complete all elements of the questionnaire (n=199+86-12=273). EQ-5D-5L was analysed using ordinal logistic regression based on the categorised score by the following categories: 1, ≥0.8 to <1, ≥0.5 to <0.8 and <0.5

**Abbreviations:** DVA= Distance visual acuity, MD= Mean difference, HR= Hazard ratio, OR= Odds ratio, CI= Confidence interval, EQ-5D-5L=EuroQoL,five dimension five level questionnaire
