# Supplementary material for: Long-term Visual Outcomes after Release from Protocol in Patients who Participated in the Inhibition of VEGF in Age-related Choroidal Neovascularisation (IVAN) Trial
Source: Ophthalmology. 2020 Sep;127(9):1191–200. doi: 10.1016/j.ophtha.2020.03.020 (PMC7471837; doi:10.1016/j.ophtha.2020.03.020)
Supplement: Supplementary material [file mmc17.docx]

Statistical methods1– additional information

Following release from protocol, the rate of change of DVA per year and factors associated with the rate of change were estimated from a mixed effects regression model with time included as a random effect. This allowed for patient level intercepts and slopes to vary. An interaction term between time (within-person) and the fixed (between-person) variables was added to investigate whether the rates of change of DVA differed between groups of patients with different characteristics. A forward stepwise approach was taken to select interactions to be included in the final model, using likelihood ratio tests to compare nested models with a 10% significance level. If the interaction was not statistically significant an overall effect is reported. If the interaction was statistically significant, variation in the rate of change of DVA with the covariate is reported.

Missing covariates were imputed using multiple imputation methods. Twelve imputed data sets were generated, and the results were combined using Rubin’s rule^1^. Assumptions underpinning the statistical models were checked using standard methods (e.g. residual plots). In all tables missing data are described in footnotes. A 5% significance level (two-sided) was used unless otherwise stated.

Three sensitivity analyses were carried out to test the robustness of the estimates from the model because the model: fitted injection rate in the second year of the trial (when participants were treated according to their continuous or discontinuous allocation) as the covariate for DVA in the first year of follow-up; included DVA measurements only up to the end of study monitoring (see Methods); and imputed covariates where these were missing.

DVA measured using Snellen fraction, LogMAR and no letters read (count fingers/hand movements/perception of light) were mapped to ETDRS letters (supplementary Table S1).

**Analyses of outcomes by randomized allocations**

Demographics were summarised by the original randomized drug and treatment regimen allocations. Standardized mean differences were calculated to quantify imbalances in baseline characteristics by the original trial allocations for the participants in the follow-up cohort. The effects of the original randomized allocations on the most recent DVA/BCVA in the study eye and survival since IVAN entry were assessed using linear regression and Cox proportional-hazards regression respectively. Survivors were censored at the date of their last recorded attended visit. Analyses were adjusted for centre size (seven strata as per the IVAN trial^2^). Linear regression could not be used to assess the effect of the randomised allocations on EQ-5D-5L as the assumptions of linear regression were not met. Therefore, a graded categorical scale was used to classify participants based on the index score: 1 indicated no problem in any dimension; 0.80 to 0.99 indicated a moderate problem, in only one dimension; 0.5 to 0.79 two or more moderate problems in any dimension; and <0.5 at least one extreme problem^3^. Ordinal logistic regression was then used.

**References**

1. Rubin D. Multiple Imputation for Nonresponse in Surveys. New York: John Wiley and Sons; 2004.

2. Chakravarthy U, Harding SP, Rogers CA, Downes SM, Lotery AJ, Culliford LA, et al. Alternative treatments to inhibit VEGF in age-related choroidal neovascularisation: 2-year findings of the IVAN randomised controlled trial. Lancet (London, England). 2013;382(9900):1258-67.

3. Cavrini G. A quantile regression approach for modelling a Health-Related Quality of Life Measure. 2010. 2010;70(3):19 %J Statistica.
